# Araneae of Canada

**DOI:** 10.3897/zookeys.819.26391

**Published:** 2019-01-24

**Authors:** Robb Bennett, Gergin Blagoev, Claudia Copley

**Affiliations:** 1 Department of Entomology, Natural History Section, Royal British Columbia Museum, 675 Belleville Street, Victoria, British Columbia, V8W 9W2, Canada Royal British Columbia Museum Victoria Canada; 2 Centre for Biodiversity Genomics, University of Guelph, 579 Gordon Street, Guelph, Ontario, N1G 2W1, Canada University of Guelph Guelph Canada

**Keywords:** Araneae, BINs, biodiversity assessment, Biota of Canada, checklist, classification, DNA barcoding, faunistics, spiders

## Abstract

In 1979 nearly 1400 spider species in 32 families either had been recorded (1249) or were believed to occur (~140) in Canada. Twenty years later, although significant progress had been made in survey efforts in some regions, Canada’s spider inventory had only increased by approximately 7% to roughly 1500 species known or expected to occur. The family count had increased to 38 but only two additions were truly novel (five family additions and one family deletion were the result of advances in family-level systematics). The first comprehensive taxonomic checklist of Canadian spider species was published in 2010 documenting the regional distributions of 1376 species representing 42 families (three novel since 1999). From 2010 through 2017 new national records steadily accumulated resulting in the current (2018) Canadian inventory of 1477 species classified in 45 families (one novel since 2010). Although there has been close to a 20% increase in the number of spider species recorded in Canada since 1979, much greater increases have occurred in some of the regional species checklists, indicating increasing knowledge of the regional distribution of species previously recorded elsewhere in Canada. For example the regional checklists for Newfoundland, British Columbia, and Prince Edward Island have increased by 69%, 339%, and 520%, respectively. The national and regional increases reflect significant advances in the first two decades of the 21^st^ Century in spider faunistics research in previously under-sampled habitats and regions and the development of molecular techniques and consequent barcoding of spiders. Of the 1477 species recorded in Canada, 92% have been successfully DNA barcoded resulting in 1623 unique Barcode Index Numbers (BINs). At least 25 of the BINs are associated with relatively easily distinguished but undescribed morpho-species. The majority, however, appear to indicate the existence of many cryptic species within Canada’s known spider fauna. These data, coupled with the fact that novel Canadian or even Nearctic spider species records (including of undescribed species) continue to accumulate annually (especially in habitat-diverse regions such as British Columbia), suggest that Canada’s tally of spider species may approach or even exceed 1800.

## Introduction

Canadian national spider faunistics work commenced with Emerton’s (1920) initial listing of the names of and basic locality data for the spider species then known to occur in Canada. Emerton’s list was far from complete, reflecting the scant knowledge of Canada’s spider fauna at that time. Nearly 60 years later the accumulation of sufficient data allowed production of the first reasonably comprehensive faunistic overview of the spiders of Canada (Dondale 1979). Subsequently, a series of increasingly detailed studies of the fauna has significantly improved the situation. Dondale (1979) provided the initial faunistics framework: a family-level table outlining general distributions and estimated species numbers for each, accompanied by a brief introduction to spiders, Canada’s spider fauna, and basic biology of the major families.

Twenty years later, Bennett (1999) supplied a similar table with revised family-level nomenclature and species statistics, as well as more detailed family-level biology and comments on the then-current state of araneology in Canada. Subsequently, Paquin et al. (2010) published a checklist of all spider species found in Canada up to that date including species-level provincial and territorial distributional data, tables with summary family-level statistics, and a list of introduced species.

Most recently, the Canadian Endangered Species Conservation Council (2011, 2016) provided updated species-level checklists, including novel records and nomenclatural changes, as well as national and provincial/territorial conservation ranks for all species using NatureServe distribution and abundance analysis methodology (see Faber-Langendoen et al. (2012) for a description of NatureServe’s methodology). Since then, two of us have maintained and updated the Canadian national and regional checklists (R Bennett and G Blagoev unpubl. data, summarised in Table 1). Unsurprisingly, the known Canadian spider fauna has been (and continues to be) dominated by species of Linyphiidae (Table 2).

Prior to 1979, regional spider species checklists existed for only two Canadian regions (Table 3): British Columbia (Thorne 1967, Bragg and Leech 1972) and the island of Newfoundland (Hackman 1954). Species-level data now exist for all Canadian provinces and territories (Table 3; Paquin et al. 2010) and a majority of these have series of three or more checklists showing increases in the accumulation of novel jurisdictional species records of greater than 20% (Alberta, Manitoba, Northwest Territories, Québec, and Yukon), nearly 70% (island of Newfoundland), to well over 300% (British Columbia) or 500% (Prince Edward Island).

**Table 1.** Census of Araneae in Canada. Data as of March 2018 (R Bennett and G Blagoev unpubl. data). Primary references for all family-level and lower taxa are in the World Spider Catalog (2018) and Ubick et al. (2017).

Taxon^1^	No. species currently known from Canada^2^	No. BINs^3^ available for Canadian species	Distribution by ecozone^4^
**Suborder Opisthothelae**			
**Infraorder Mygalomorphae**			
**Superfamily Atypoidea**			
Mecicobothriidae	1	1	Pacific Maritime
Antrodiaetidae	4	5	Pacific Maritime, southern Montane Cordillera
Atypidae	1	1	Mixedwood Plains
“**Dipluroidea**”			
Dipluridae	1	0	southern Montane Cordillera
**Infraorder Araneomorphae**			
**Division Synspermiata^5^**			
**Superfamily Dysderoidea**			
Segestriidae	1	1	southern Pacific Maritime
Dysderidae	1 (1)	2	southern Pacific Maritime, Mixedwood Plains; synanthropic
Oonopidae	2 (2)	0	southern Pacific Maritime, Mixedwood Plains; synanthropic
**Superfamily Scytodoidea**			
Scytodidae	2 (2)	1	Mixedwood Plains; synanthropic
“**Pholcidoidea**”			
Telemidae	1	3	Pacific Maritime, Montane Cordillera
Pholcidae	5 (3)	7	southern Montane Cordillera (native spp.); all southern ecozones (synanthropic spp.)
**Division Entelegynae**			
**Superfamily Araneoidea**			
Theridiidae	107 (19)	107	all ecozones
Anapidae	1	1	southern Pacific Maritime
Theridiosomatidae	1	2	Boreal Shield, Newfoundland Boreal, Mixedwood Plains, Atlantic Maritime
Araneidae	67 (5)	79	all ecozones
Pimoidae	3	5	Pacific Maritime, southern Montane Cordillera
Linyphiidae	569 (12)	624	all ecozones
Nesticidae	3 (1)	3	Pacific Maritime (native sp.), Mixedwood Plains (native and synanthropic spp.)
Mysmenidae	3	2	southern Pacific Maritime, southeastern Boreal Shield, and Mixedwood Plains
Mimetidae	9 (1)	7	all ecozones except Arctic
Tetragnathidae	26 (2)	72	all ecozones
**Superfamily Oecobioidea**			
Oecobiidae	2 (2)	0	southern Pacific Maritime, Mixedwood Plains; synanthropic
“**Uloboroidea**”			
Uloboridae	5 (1)	6	all ecozones south of Boreal Shield, Newfoundland Boreal, and Boreal Cordillera
“**Titanoecoidea**”			
Titanoecidae	4	4	all inland ecozones
**Superfamily Zodarioidea^6^**			
Zodariidae	1 (1)	1	southern Pacific Maritime, Mixedwood Plains; synanthropic
“**Amaurobioidea**”			
Amaurobiidae	14 (2)	13	all ecozones
“**Desoidea**”			
Desidae	1 (1)	0	Boreal Plains; synanthropic
**Superfamily Agelenoidea^7^**			
Dictynidae	62 (2)	79	all ecozones
Cybaeidae	22	24	Pacific Maritime, Montane Cordillera, Boreal Shield
Hahniidae	21	34	all ecozones
Agelenidae	20 (4)	15	all ecozones
**Superfamily Lycosoidea**			
Oxyopidae	2	3	all southern ecozones
Thomisidae	66 (2)	81	all ecozones
Pisauridae	7	8	all ecozones
Lycosidae	104 (2)	103	all ecozones
“**Salticoidea”^8^**			
Salticidae	124 (7)	126	all ecozones
Philodromidae	47 (3)	54	all ecozones
Corinnidae	8	6	all southern ecozones
Eutichuridae	3 (1)	4	all southern ecozones
Miturgidae	1	1	southern Pacific Maritime, Montane Cordillera
“**Anyphaenoidea**”			
Anyphaenidae	7	8	all southern ecozones
Clubionidae	34 (2)	35	all ecozones
“**Liocranoidea**”			
Liocranidae	3	3	all ecozones
“**Trochanterioidea**”			
Trachelidae	3	6	southern Pacific Maritime, Mixedwood Plains, Atlantic Maritime
Phrurolithidae	16 (1)	14	all southern ecozones
Gnaphosidae	92 (3)	72	all ecozones including southern Arctic
**Total**	**1477 (81)**	**1623**	

**^1^** Classification follows Wheeler et al. (2017); taxon names in quotation marks are informal. **^2^**Parentheses enclose numbers of non-native species included in the total. **^3^**Barcode Index Number (Ratnasingham and Hebert 2013). ^4^See figure 1 in Langor (2019) for a map of ecozones. **^5^**Haplogynae of older classifications. **^6^**Zodarioidea and all subsequent groups = “RTA Clade”. **^7^**Dictynoidea of older classifications. **^8^**“Salticoidea” and all subsequent groups = “Dionycha Clade” (within RTA Clade).

**Table 2.** Changes in numbers of species for selected spider families in Canada (1979–2018).

Family	1979^1^	1999^2^	2010^3^	2016^4,5^	2018^6^	Increase (decrease)
Theridiidae	93	100	99	102	107	14
Araneidae	69	74	57	58	67	(2)
Linyphiidae	445	>500	527	542	569	124
Tetragnathidae	21	23	25	25	26	5
Thomisidae	63	68	65	65	66	3
Lycosidae	90	110	101	99	104	14
Salticidae	100	110	108	110	124	24
Philodromidae	47	47	48	46	47	0
Gnaphosidae	63	~100	88	90	92	29

^1^Dondale 1979. ^2^Bennett 1999. ^3^Paquin et al. 2010. ^4^Canadian Endangered Species Conservation Council 2016. ^5^ Based on 2013 data. ^6^ R Bennett and G Blagoev unpubl. data.

**Table 3.** Changes in Canadian national, provincial, and territorial spider species numbers over time and percent increase since 2010.

Time period	AB	BC	LB	MB	NB	NF^1^	NS	NT	NU	ON	PE	QC	SK	YT	CAN
1950–1979		212^6^				216^20^									
		259^7^													1249^27^
1980–1999		433^8^													
		570^9^		483^18^								549^23^		297^26^	~1400^28^
2000–2010		653^10^										620^24^			
	527^2^	656^11^				363^21^						654^25^			
	601^3^	700^12^	124^3^	531^3^	379^3^	363^3^	437^3^	267^3^	71^3^	746^3^	38^3^	677^3^	489^3^	335^3^	1376^3^
2011–2018		729^13^													**
		780^14^	213^17^												
	628^4^	859^15^	212^4^	605^4^	390^4^	364^4^	446^4^	321^4^	96^4^	757^4^	44^4^	666^4^	490^4^	357^4^	1399^4^
	656^5^	877^16^	*	593^19^	416^19^	*	472^19^	325^19^	98^19^	813^19^	198^22^	691^19^	507^19^	366^19^	1477^19^
% increase since 2010	10%	25%	71%	12%	10%	<1%	8%	22%	4%	9%	521%	2%	4%	9%	7%

^1^Excluding Labrador. ^2^Buckle and Holmberg in Pickavance and Dondale 2005. ^3^Paquin et al. 2010. ^4^Canadian Endangered Species Conservation Council 2016. ^5^R Bennett and G Blagoev unpubl. data. ^6^Thorne 1967. ^7^Bragg and Leech 1972. ^8–9^West et al. 1984, 1988. ^10^Bennett 2001. ^11–16^Bennett et al. 2006, 2010, 2012, 2014, 2017, unpubl. data. ^17^Perry et al. 2014, ^18^Aitchison-Benell and Dondale 1990. ^19^R Bennett and G Blagoev unpubl. data. ^20^Hackman 1954 as revised in Pickavance and Dondale 2005. ^21^Pickavance and Dondale 2005. ^22^Bowden et al. in press. ^23^Bélanger and Hutchinson 1992. ^24^Paquin and Dupérré 2003. ^25^Paquin and Dupérré 2006. ^26^Dondale et al. 1997. ^27^Dondale 1979 as revised this study. ^28^Bennett 1999. * Current (2018) data combine LB and NF together (total 434 species) and are not easily separable. Increase since 2010 for LB and NF combined = 18%. ** National and provincial/territorial species count data in Canadian Endangered Species Conservation Council (2011) are essentially unchanged from those in Paquin et al. 2010 and are not recorded here.

Novel species records, including of undescribed species, continue to accumulate annually in Canada especially through the work of the University of Guelph’s Centre for Biodiversity Genomics (CBG) at the national level and, regionally, where intensive spider inventory work is on-going, e.g., in British Columbia through the efforts of the Royal British Columbia Museum (RBCM). Ideally, faunistics research generates sufficient distribution and abundance data to allow for conservation ranking analyses. For more than one third of Canada’s recorded spider species, however, only simple presence data exists and conservation ranks remain undetermined for those species. It is clear that much work remains to be done on Canadian spider faunistics. Canada’s national and provincial/territorial spider species checklists will continue to grow, our knowledge of Canadian spider morphological and molecular taxonomy will continue to improve, and accumulating inventory data will allow for conservation ranking of a significant proportion of Canada’s currently unranked spider fauna.

Here we present an overview of Canadian spider faunistics work from 1979 to 2018, focusing on the accumulation of national and regional species-level data. Classification follows Wheeler et al. (2017); nomenclature follows the World Spider Catalog (2018). Standard postal abbreviations are used for Canada’s provinces and territories except for LB and NF, which refer to mainland Labrador and the island of Newfoundland, respectively: AB – Alberta, BC – British Columbia, MB – Manitoba, NB – New Brunswick, NS – Nova Scotia, NT – Northwest Territories, NU – Nunavut, ON – Ontario, PE – Prince Edward Island, QC – Québec, SK – Saskatchewan, and YT – Yukon.

## In the beginning (Dondale 1979)

Although Emerton (1920) presented a cursory list, including basic locality data, of about 340 spider species then known to occur in Canada, the first serious summary of faunistics of Canada’s spider species was published by Dondale (1979). The single table in that paper provided the faunistics framework for future studies of Canada’s spider fauna: a list of the 32 families recorded to that date accompanied, for each family, with general Canadian ecological distribution data and the number of species known in Canada. Loxoscelidae (now Sicariidae), a 33^rd^ family listed in the table, was then unrecorded in Canada and has not become established subsequently. Dondale predicted a total of about 1400 species for Canada’s spider fauna: 1249 recorded (Table 3; number here revised down from 1256 because of enumeration errors in Dipluridae, Antrodiaetidae, Dysderidae, and Segestriidae (CD Dondale pers. comm.)) with a further 144 either known but undescribed or expected but not yet recorded.

Then, as is typical for the Holarctic region (Coddington and Levi 1991), entelegyne taxa, especially Linyphiidae (including Pimoidae and Dondale’s “Erigonidae”), dominated the known Canadian spider fauna (Table 2): 445 of the species recorded (as well as more than half of the expected/undescribed species). Globally common and taxonomically fairly well-studied families accounted for a further 546 species (Table 2) representing, in combination with Linyphiidae, nearly 80% of the Canadian fauna. Species then classified in several other entelegyne families (especially Agelenidae, Dictynidae, Hahniidae, and Clubionidae) covered an additional approximately 15% but, because those families have proven to be non-monophyletic and subsequent species transfers have complicated the picture, family-level data for them from Dondale (1979) are not presented here. Families typical of warmer climates and/or austral regions, especially mygalomorph and synspermiate taxa (Dondale 1979), as well as a handful of entelegyne taxa, were represented by about two dozen species.

In 1979, only two Canadian jurisdictions, BC and NF (Table 3), had spider checklists, and these were not comprehensive for either province. Thorne (1967) listed 212 species in 20 families for BC, subsequently upgraded to 259 species by Bragg and Leech (1972). Thorne noted BC’s spider fauna was poorly known and implied that significant increases to the provincial checklist would result from better knowledge, in general, of the province’s northern interior and northern and eastern border areas and, in particular, of the province’s erigonine linyphiid fauna (see Bennett (2001) for a brief history of araneological work in BC from the late 1800s to 2001). Hackman (1954) documented 216 species (originally 220, revised down by Pickavance and Dondale (2005)) in 19 families for NF. Because his study was based almost entirely upon only two sets of collections made over two summers (1949, 1951) by visiting scientists from Fennoscandia, it is clear that Hackman’s (1954) checklist was not comprehensive.

## Making progress (Bennett 1999)

Twenty years after Dondale’s (1979) treatment of the national fauna, Bennett (1999) updated Canadian family-level spider species statistics. Estimates, rather than exact counts, were made for most of the larger entelegyne families (e.g., Dictynidae – “75–80”, Linyphiidae – “> 500”) and the total number of species then recorded in Canada was estimated to be approximately 1400 (Table 3). Bennett (1999) speculated that the species count could reach 1500 with concerted inventory work in particular habitats, especially forest floors and canopies. Increases in species counts (Table 2) were, as expected, primarily in Linyphiidae (more than 50 species added) but also in groups that had been subject to significant taxonomic revision since 1979, such as Gnaphosidae (about 40 species added) and Lycosidae (20 species). Species counts for other families were relatively unchanged.

Although Bennett’s (1999) checklist recorded 38 spider families in the Canadian fauna, six more than reported by Dondale (1979), only two families, Zoridae and Mysmenidae, were truly novel additions through the discovery of populations in BC of species known to be relatively widespread in the western United States. Five other family additions were not novel but the result, rather, of advances in spider systematics and classification: in Dondale (1979)Cybaeidae was classified in Agelenidae, Liocranidae and Corinnidae in Clubionidae, Titanoecidae in Amaurobiidae, and Pimoidae in Linyphiidae. Dondale’s (1979)Erigonidae was not included in Bennett (1999) and subsequent lists because of its reclassification as a subfamily of Linyphiidae.

In the 1980s and 1990s, considerable progress was made on Canada’s regional spider checklists (Table 3), a reflection of significant spider inventory work in some regions. BC’s spider species count more than doubled to 570 (West et al. 1984, 1988) and initial checklists were produced for MB (483 species; Aitchison-Benell and Dondale 1990), QC (549; Bélanger and Hutchinson 1992), and YT (297; Dondale et al. 1997). As with the earlier checklists for BC and NF, the regional checklists of the 1980s and 1990s were still far from complete and major changes would be made through the next couple of decades.

## The recent past (Paquin et al. 2010)

Early in the first decade of the 21^st^ Century, DJ Buckle (pers. comm.) began compiling a checklist including the names and basic regional (Canadian provinces and territories and/or Alaska) presence data for all spider species then recorded in Canada and Alaska. The database was subsequently published (Paquin et al. 2010), making widely available for the first time a reasonably comprehensive Canadian national and regional species-level checklist. Thirty-six of the species were known from Alaska but not from anywhere in Canada (one species in each of Clubionidae and Dictynidae, three in Lycosidae, and 31 in Linyphiidae) and one South American species (Sicariidae, see above) was not established in Canada then and has not become so subsequently. Removing those 37 species from consideration, the 2010 checklist recorded 1376 species (Table 3) representing 42 families in one or more of Canada’s provinces and territories. Although this species count was not appreciably different from Bennett (1999), four families were newly recorded by Paquin et al. (2010). Three of these families were first Canadian records of non-native species in Amphinectidae (one species, now classified in Desidae), Oonopidae (two species), and Zodariidae (one species). The addition of a fourth family, Miturgidae, was the result of advances in systematics (transfer of two well-known species from Clubionidae). Among the new introductions, the status of the desid and the oonopids is uncertain. No new records are known since the first (and only) record of the desid in AB in 1991. One of the oonopids has been collected only once, albeit from a natural (but disturbed) outdoor habitat in Victoria BC; the other species is known from a substantial indoor population in Toronto ON (Platnick et al. 2012) and is probably established there. The zodariid is almost certainly an established member of the Canadian fauna; it is now known from relatively natural habitats in southern localities in three provinces (R Bennett and G Blagoev unpubl. data) and appears to be well established in the northern United States (Ramseyer 2016). As in Bennett (1999), family-level species counts in Paquin et al. (2010) showed substantial increases only in Linyphiidae, up about 30 since 1999 (Table 2).

In addition to revising the species names and count data for regions with existing species-level checklists (AB, BC, MB, NF, QC, and YT), a major benefit of Paquin et al. (2010) was the provision of species-level data for those Canadian regions where spider species checklists had not previously existed (Table 3). The low species counts for PE and NU reflected the fact that no serious spider inventory work had yet been done in PE (Bowden et al. in press) and very little in NU (e.g., Leech 1966). The species numbers recorded in 2010 for the remaining “first-time” regions are an indication of substantial spider inventory work in those regions, largely led by CD Dondale and JH Redner of the Canadian National Collections (CNC) but also with significant input of specimens and data from CNC entomologists, Canadian Forest Service researchers, and non-government arachnologists such as DJ Buckle, R Holmberg, and RE Leech.

In the first decade of the 21^st^ Century, Canadian regional spider faunistics work continued to be especially active in BC and QC where, by 2010, both provinces had recorded increases of about 130 species to 700 and 677, respectively (Table 3). In addition, a short burst of intensive spider faunistics work in NF resulted in an increase of more than 150 species for a total of 363 species on the island’s spider checklist (Pickavance and Dondale 2005). Based primarily on large numbers of spider specimens collected during research in the 1990s in localised areas of southern and central BC led by DCA Blades (Blades and Maier 1996), GGE Scudder (unpublished), N Winchester (Copley 2010, Copley and Winchester 2010), and BS Lindgren and J Lemieux (Lindgren et al. 1999), Bennett (2001) and Bennett et al. (2006) updated, respectively, the BC spider species count and the checklist of names and locality data. Family and species-level nomenclature in Bennett et al. (2006) were in agreement with current World Spider Catalog (Platnick 2006) standards, thus simplifying tracking of taxonomic synonyms and other nomenclatural changes in preceding and subsequent BC spider checklists. Two of us (R Bennett and C Copley) and D Copley used locality data in Bennett et al. (2006) to map unsampled or poorly sampled regions and habitats in BC and plan a series of annual field surveys, primarily targeting alpine and subalpine habitats across the province, to address the knowledge gap. With support from the RBCM and a wide range of collaborators, these surveys commenced in 2008 and the first two field seasons resulted in the addition of more than 40 species to the known spider fauna of BC (Bennett et al. 2010), some of which were new records for Canada or even the Nearctic. In QC, the significant efforts of the team of Paquin and Dupérré (2003, 2006) in the field (as well as in examining specimens in the CNC for overlooked new records) were largely responsible for the increases in the list of spider species known in QC.

## The current era and into the future (Canadian Endangered Species Conservation Council 2016 and the rise of BINs)

In 2010, using a draft version of the species list in Paquin et al. (2010) as their baseline, attendees at an ad hoc workshop organised by NatureServe Canada produced an initial consensus-based assessment of the national and regional conservation status of all spider species recorded in Canada (Canadian Endangered Species Conservation Council 2011). In 2013, the Canadian Wildlife Service (Environment Canada) began a comprehensive reanalysis of the conservation status data using NatureServe methodology (Faber-Langendoen et al. 2012) and incorporating all available spider species distribution and abundance data as well as the significant number of novel species records that had accumulated since 2010. The results of the analysis included updated national and regional checklists recording 1399 spider species (Table 3) representing 42 families (data current to 2013; Canadian Endangered Species Conservation Council 2016) and, in 2018, this is the contemporary published resource for species-level faunistic data for Canadian spiders. Family number remained unchanged from 2010 although Zoridae was incorporated into Miturgidae and Anapidae was newly recorded for Canada. Notably, the anapid record, *Comaromamendocino* (Levi), collected in BC, is one of the rarest of all native spider species in North America (see Lopardo and Coddington 2017). New records of Linyphiidae accounted for two thirds of the relatively minor increases in Canada’s family-level spider species counts reported in the 2016 publication (Table 2).

Since 2013, i.e., data incorporated in Canadian Endangered Species Conservation Council (2016), new national records have accumulated rapidly. Seventy-eight new species records have been documented from across Canada (with the majority from BC) resulting in the current Canadian inventory of 1477 spider species in 45 families (Table 1). Linyphiid species account for more than one third (27) of the new records (Table 2) but some families which had not seen much change for several decades also show substantial increases, for example araneid and salticid species counts have increased by 9 and 23 (Table 2), respectively. All three of the new family names in Table 1 are the result of recent classification changes: Eutichuridae was previously classified in Miturgidae while Phrurolithidae and Trachelidae were extracted from Corinnidae.

As with previous checklists, the increased species counts largely reflect the continuation of serious regional spider inventory work. However, molecular data have become increasingly important in Canadian spider faunistics and systematics, and the current Canadian spider species checklist (R Bennett and G Blagoev unpubl. data) is the first to benefit from the use of molecular data. The molecular work, led by the CBG, has combined laboratory work with comprehensive sampling of arthropod specimens in a wide variety of often difficult-to-access and/or previously poorly known habitats in Canada (deWaard et al. 2017). The RBCM has closely collaborated with the CBG since 2012, resulting in large amounts of novel regional as well as national species-level data (Bennett et al. 2017). In particular, molecular data have been critical in the identification of specimens of described species that previously were unidentifiable (or easily misidentified) because of the lack of good morphology-based diagnostic images or reliably identified voucher specimens (e.g., various linyphiid taxa).

More than 50,000 Canadian spider specimens have been analysed in the course of the barcoding work, representing 92% of the 1477 spider species currently recorded in Canada (Table 1; R Bennett and G Blagoev unpubl. data) and generating 1623 individual Barcode Index Numbers (BINs) (Ratnasingham and Hebert 2013). Unsurprisingly, because nearly 40% of all Canadian spider species are linyphiids, nearly one third of the sequenced specimens are Linyphiidae and those specimens account for nearly 40% of the resultant BINs. BINs in excess of known species-level taxon numbers usually indicate the potential for undescribed species and complexes of cryptic species. This is particularly evident among some smaller families (e.g., Telemidae, Theridiosomatidae, Tetragnathidae, and Trachelidae) which have generated 2–3 times as many distinct BINs as there are named species in the Canadian fauna of those families. Such BIN disparity can be particularly striking for individual species. For example, 18 BINs are associated with a single species, *Tetragnathaversicolor* Walckenaer. For this species, the disparity is not a big surprise: *T.versicolor* is well known for its morphological variability and probable masking of a cryptic species complex (Levi 1981). Similarly, 19 and 25 unique BINs have been generated from specimens of *Evarchahoyi* (Peckham and Peckham) (Salticidae) and *Grammonotaangusta* Dondale (Linyphiidae), respectively.

The accumulation of new provincial/territorial records across Canada has been especially impressive for some jurisdictions (Table 3). Most dramatically, the species count for PE increased by 521% between 2010 and 2018. This was almost entirely because of the combination of data accumulated through CBG’s national barcoding project (Blagoev et al. 2016, deWaard et al. 2017) and a “BioBlitz” in 2015 targeting PE’s previously poorly known spider fauna (Bowden et al. in press). (BioBlitzes, a relatively new faunistics tool, can be important sources of novel data. For example, in 2014 and 2015 the participation of a team of arachnologists in two BioBlitzes in the small area of Ojibway Park in Windsor ON resulted in the addition of twenty new species records (including six new genus records) to the checklist of Canadian spiders.) The annual intensive surveys of alpine and subalpine habitats in BC begun by the RBCM in 2008 have continued and, along with significant input of specimens and data from a wide variety of collaborators, have resulted in a series of provincial checklists documenting a 25% increase in BC’s recorded spider fauna between 2010 and the end of the 2017 field season (Bennett et al. 2012, 2014, 2017, unpubl. data). Spider faunistics in northern Canada benefited from intensive surveys at arctic and subarctic sites by S Loboda and C Buddle (McGill University), R Wisseman (Aquatic Biology Associates, Inc., Corvallis), Perry et al. (2014), the CBG, and others; spider species number increases since 2010 of 71% (124 to 212) and 22% (267 to 325) for LB and NT, respectively, are largely the result of that work. The CBG has also continued to support regional work led by others, such as in BC and PE. This has been mutually beneficial, helping the CBG to complete its library of BINs for Canadian spider taxa and the provinces and territories to achieve a more complete understanding of their regional spider faunas.

## Summary (and some predictions for the future)

The rate of increase in Canada’s spider species count shows little sign of slowing down. In 2018 the count is well beyond the approximately 1400 predicted by Dondale (1979) and is rapidly closing in on Bennett’s (1999) estimate of about 1500. For example, consider the case of BC, which has a longer and more consistent history of spider faunistics research than do other regions of Canada (Table 3) and is the nation’s most ecologically diverse province (Morrison and Turner 1994). On average, more than 20 species (primarily linyphiids) have been added annually to the BC regional checklist since 2006 (many of the new records are the result of the RBCM’s on-going collaboration with the CBG) and more than 70 of the additions to the BC checklist are of species not previously recorded in Canada and, in some instances, the Nearctic (Bennett et al. 2016, 2017). Although the inventory work conducted annually since 2008 by RBCM researchers and their colleagues has resulted in a major increase in the area of BC surveyed for spiders (see fig. 1 in Bennett et al. 2017), habitats in much of the province remain unsurveyed, especially in the province’s Boreal Cordillera, Taiga and Boreal Plains, northern Montane Cordillera, and central Pacific Maritime ecozones. Nearly two thirds of all spider species recorded in Canada occur in BC (Table 3). The Nearctic region is home to close to 3800 spider species (Cushing 2017), nearly one quarter of which have been recorded in BC. Clearly, BC is an important region for Nearctic spider diversity and, assuming the RBCM-led work continues, the BC spider species count eventually is likely to exceed 1000, as first predicted by Bennett et al. (2012). Some proportion of those species will be novel Canadian or even Nearctic records. Although other regions of Canada lack BC’s complex ecological diversity, with the exception of PE, all have vast amounts of habitat in which the spider fauna has never been surveyed (or only poorly so). Canada’s boreal forest, southern hardwood forest, alpine/subalpine, and subarctic tundra habitats are excellent candidates for the discovery of novel regional and national species records given sufficient search effort. As well, provinces sharing borders and particular terrestrial habitats with the United States (e.g., BC’s dry southern interior valleys; the southern grasslands of AB, SK, and MB; and southern ON’s Carolinian forests and tallgrass prairies) have been the source of novel national species records historically (usually northern range extensions of southern species) and likely will continue to be so in a warming climate.

Finally, DNA barcoding has proven to be an effective complement to classic morphological techniques for identifying spiders and contributing to the advancement of Canadian spider faunistics (Barrett and Hebert 2005, Robinson et al. 2009, Blagoev et al. 2013, 2016, Blagoev and Dondale 2014). At this time the CBG’s reference library, BOLD (Ratnasingham and Hebert 2007), contains DNA barcodes for 92% of Canada’s 1477 known spider species. Many BINs, appear to reflect cryptic species complexes within well-known taxa (e.g., the example provided by *Tetragnathaversicolor*) but nearly 60 BINs are associated with Canadian taxa that remain unidentified for a variety of reasons. These have interim, temporary names in BOLD and eventually may be matched to named taxa pending sufficient laboratory detective work or future sequence matching. At least 25 of the BINs, however, are associated with species that are clearly new to science – unnamed and undescribed but morphologically easily discernible. For example, during the period 2014 to 2017 many hundreds of specimens of an unidentifiable but morphologically distinctive species of *Perro* Tanasevitch (Linyphiidae) were collected at alpine localities in northern BC and southern YT. DNA barcoding and associated taxonomic detective work at the CBG confirmed that this taxon, although morphologically distinctive and common and abundant in relevant habitat, is a Beringian species new to science. Another example is provided by the first Nearctic records of the Palaearctic genus *Mughiphantes* Saaristo and Tanasevitch (Linyphiidae), an undescribed species initially revealed in 2009 by barcoding of originally unidentifiable specimens from Churchill MB (Blagoev et al. 2013). Subsequent barcoding of further unidentified specimens from other localities has demonstrated that the species is relatively common and widespread in subarctic habitats in AB, BC, LB, MB, NF, QC, and YT.

Future additions to the checklist of Canadian spiders will likely follow the historical precedent shown in Table 2: dominance by linyphiids augmented by new data from difficult groups (e.g., salticids) or emerging from formal taxonomic revisions (e.g., such as occurred with gnaphosids and lycosids between 1979 and 1999). The most recent published estimate for the “actual total” number of spider species in the Canadian fauna (Paquin et al. 2010) is approximately 1720. This is probably a reasonable estimate, given the continuing accumulation of substantial numbers of named species to the checklist of the Canadian spider fauna and of species new to science and awaiting description prior to their addition to the national checklist. However, if most of the excess BINs reflect real but cryptic species, it will not be surprising to see the species count approach or exceed 1800. Tune in again in 20 or 30 years…
